# Clinique Chirurgicale de l'Hôpital de la Pitié

**Published:** 1843-04-01

**Authors:** 


### Clinique Chirurgicale de l'Hôpital de la Pitie. Par *J. Lisfranc*. Tome Second, 8vo. pp. 738. Paris, Bechet, 1842.

Our readers will doubtless learn, with much regret, that the distinguished author of the present volume has been for some time past, and still is, labouring under a very distressing and obstinate malady. It has hitherto, we are sorry to say, baffled the best efforts of his professional brethren in his own country; and, from this circumstance alone, there is but too much reason to fear that it has already taken so deep a root in his system, that it may defy every remedial resource of the healing art. At the close of the present article, we shall state at full length the opinion which we have formed of the malady that has so long afflicted M. *Lisfranc*—perhaps we should rather say his family and friends—and explain the course of treatment which we have recommended to be tried. Meanwhile we proceed with our duty, as Reviewers, to examine his last published volume—rich in many novel views on pathology, and containing much curious information. We have rarely met with a work that is so truly a transcript of an author's character as the present one.

In the first place, let the title of the volume be remarked :—" Clinique Chirurgicale de l'Hôpital de la Pitié," or, in other words, bed-side reports of surgical cases in La Pitié Hospital, with comments thereon.

The table of contents presents us with a catalogue of the following subjects ;—

Tumors of the mammæ—surgical anatomy of the female organs of generation—pruritus of the vulva—excessive sensibility of the generative organs—diseases of the womb—manual examination and the use of the speculum—leucorrhœa, chlorosis, menstruation, amenorrhœa, menorrhagia—prolapsus of the vagina, nymphomania, hysteria, metritis, hypertrophy and enlargement of the womb, &c. &c.

We think we hear some one saying, what the deuce have most of these subjects to do with clinical surgery? This is not very obvious, we must confess; but pray, be not hasty courteous reader; reserve thy judgment till thou hast perused with attention what M. *Lisfranc* presents to you; and, we are much mistaken indeed, if you will not then confess that a new fountain of thought, which you never dreamed of, has been opened up to you—a fountain from which your author says that you may freely draw streams of health and happiness for suffering humanity.

Without further preface, we shall proceed to a brief notice of the contents *seriatim*.

The *first* chapter on Tumors of the Mamma, although it occupies upwards of 120 pages, will not detain us long, as we have more than once of late discussed this subject at considerable length.

There is, moreover, another reason: it is no easy thing to convey to the English reader an accurate idea of a Frenchman's writings on scirrhus, and those diseases which are apt to be mistaken for it, in consequence of the indefinite meaning that is attached to that term. Scirrhus, according to almost all English writers, is a peculiar degeneration of

(usually glandular) structure, which is rarely or never dispersed, and which usually terminates in cancerous ulceration; whereas nothing is more common in French writing, than to read of *scirrhous* tumors—by which can be meant only indurated swellings—being cured.

A few gleanings, however, from M. *Lisfranc's* remarks must be given. Like every experienced surgeon, he candidly admits the exceeding difficulty of forming an accurate diagnosis in many cases; but he surely goes rather too far when he asserts that "the efforts, which pathology has hitherto made to discriminate the various kinds of mammary tumors, have been almost always both useless and even hurtful." We, however, most perfectly agree with him in the spirit of the following remarks:—

"Many persons in the present day," says he, "with the view of appearing men of genius, pretend that it is quite necessary to re-construct the edifice of science from its very foundation. Setting themselves to work, they begin to divide, sub-divide, and multiply diseases without end; and they assign to each of the multitude distinctive characters and physiognomies—which, according to them, may be readily recognised, but which in truth are utterly fallacious. These are most pernicious errors, which cause science to retrograde, instead of making any sure advances."

M. *Lisfranc* then discusses, with considerable minuteness, the various alleged causes of scirrhous development. He combats, and we have no doubt rightly, the favourite notion of many modern pathologists, that the disease is generally the product of irritative or inflammatory action. While he admits that this may be true in certain instances, he distinctly states that "he has seen not a few cases, where the mamma had become affected with genuine scirrhus, but where it was utterly impossible to recognise the operation of any obvious cause whatever. The surgeon must be on his guard not to be misled by the reports of the patients themselves, as they have always a tendency to refer their complaints to some supposed external agency."

M. *Lisfranc* is of opinion, that the universal use of stiff corsets by women, in France and other countries, has much increased the frequency of mammary tumors.

There can be very little doubt that the compression, often irregular and unequal, of the breasts by stays must aggravate, if it does not positively excite, the morbid action which gives rise to scirrhous and cancerous disease. Those, who are fat and short-necked, squeeze and bind down the mammæ as much as they can, to improve their figures; while their lean sisters, who have not much bosom, try to make one for themselves, by tightening the corset below the breasts, so as to push them upwards as high as possible.

With respect to treatment, probably most of our readers are aware that M. *Lisfranc*—although, as we have already seen, not an advocate of the inflammatory origin of scirrhus—has for many years strongly insisted on the advantages that may often be derived from diminishing vascular action in retarding the development of this disease.

The means *par excellence*, according to his experience, to prevent engorgements of the mammæ, especially in women whose catamenia used to be abundant, is unquestionably an occasional bleeding from the arm.

This may be practised once or even twice every month for some time, and then less frequently.

Many of the older surgeons approved of this plan. *Heister* knew the advantage of such treatment; for, in his Institutions of Surgery, we meet with the following passage; "*Non omissis, ubi sanguinis Copia urget, missionibus sanguinis, scarificationibus necnon venæ sectionibus, verno atque autumnali præsertim tempore instituendis. Quæ si negligantur, facillime scirrhus et carcinoma aut ulcus cancrosum redeunt.*" It would be easy to multiply authorities on the propriety of this practice. *Paré, Ledran, Petit, Pouteau,* and many other celebrated writers, have all highly praised blood-letting and the use of other parts of the antiphlogistic regime in the treatment of scirrhous affections. The occasional employment of warm baths, and the establishment of an issue in the arm are also very useful means in preventing not a few of the diseases that are apt to occur, at what has been called "the critical period" of life in woman. The repeated application of leeches around the affected mamma, followed by soothing poultices or mild lotions, the anointing of the mamma with a thick layer of mercurial ointment, and the internal administration of the *conium* are highly recommended by our author in certain cases of scirrhous disease. If the constitution of the patient be scrofulous, the ioduret of potassium should be combined with, or substituted for, the hemlock.

When all symptoms of irritative and inflammatory action are got rid of, we should then cautiously try the effects of discutient remedies. M. *Lisfranc* has been using of late years with decided advantage, he says, friction with the ointment of the ioduret of lead—one part to eight of lard:—a piece, the size of a filbert, is to be gently rubbed on the mamma every evening. A few grains of opium may be occasionally added to this ointment with advantage.*

When the engorgement is very chronic, the *pommade* recommended by *Dupuytren* may be now and then used with benefit; it consists of one part of muriate of ammonia blended with six or ten of simple mercurial ointment. Local vapour baths may be employed at the same time. M. *Lisfranc* has seen admirable effects in a few cases from the administration of calomel combined with opium, so as to affect the system. He very justly adds that, whatever remedies, whether internal or external, we may have recourse to with the view of discussing chronic scirrhous engorgements of the mammæ, we should at once suspend their use, if the slightest symptoms of local inflammatory action supervene. This is a most important rule to be attended to in the management of all diseases that have a tendency to assume a malignant character; and it is by surgeons having neglected to attend to it, that so many really useful remedies have at various times fallen into disrepute. We have plenty of remedies, if we knew but *when* and *how* to use them.

M. *Lisfranc* proceeds to discuss the propriety of excising the mamma,

---

* The ointment of the ioduret of lead is much less irritating to the skin, according to M. *Lisfranc's* experience, than that of the ioduret of potassium; and, as its discutient properties are quite as powerful, it may be preferred on most occasions.

when the use of the means now recommended has proved, after a fair trial, quite inefficacious. He protests against the ignorant haste with which many surgeons of the present day at once resort to the knife in all cases where they suspect the existence of scirrhous disease, and frankly acknowledges that he himself, as well as *Dupuytren*, Sir *A. Cooper* and others, have more than once extirpated breasts, which on dissection have proved to be very little altered. He quotes the interesting case of abscess situated in the cellular texture behind the mamma, between the gland and the pectoral muscle, recorded by Mr. Henry James Johnson, as a good instance how readily other diseases of the gland may be mistaken for scirrhus.* The success of the operation will much depend on the cause of the disease, whether this has been local or constitutional, and on the state of the system at the time, &c. It is scarcely possible, he remarks, to lay down any positive instructions to guide the surgeon in forming his opinion; as in each case there is some peculiarity, either favourable or otherwise, that must be taken into account. There is one observation made by our author at this point, that well deserves to be remembered by all the sons of Esculapius: (would that they might lay it to heart)—" the words *always* and *never*," says he, " ought to be expunged from every work on pathology or therapeutics." There is much wisdom in this sentence; the truly skilful surgeon seldom or never dogmatises. Some writers have gone so far as to condemn the operation of excising the mamma under almost all circumstances: this opinion is extravagant, and we need not therefore stop to expose its fallacy. But there is a question on which considerable difference of opinion still exists: viz. when the lymphatic glands in the neighbourhood of the diseased organ are enlarged, ought they to be always excised? and, if they cannot be reached, should the operation not be undertaken at all? Much will certainly depend upon the peculiar circumstances of each case; but it is right that every surgeon should know that the enlarged glands in the neighbourhood of a scirrhous tumor are not necessarily of a malignant nature, and that often they will subside, and even entirely disappear, after the latter has been removed. The experience of our author, as well as of *Dessault*, *Assalini*, &c. appears to be decisive on this point. Those, who wish to know the sentiments of M. *Lisfranc* on this important question at full length, will do well to read his " Considerations on Cancer," in the 1st volume of his present work.

We shall proceed therefore to the next chapter, which treats of the surgical anatomy of the female organs of generation. M. *Lisfranc* (strange to say) commences his remarks, by disclaiming all imputations of immodesty in discussing the subject in question; his only motive being, he tells us, " the sacred interest of humanity:" this is truly à la Française. As might be anticipated, the greater part of his observations are really quite unnecessary, and totally uncalled-for in a work like the present. How an author, like M. *Lisfranc*, could have for one moment dreamt of filling his book with such common-place descriptions as occupy no less than 28 pages, puzzles us not a little to conjecture. One result however of his experience may not be known to many of our readers, and we shall

---

* Vide Medico-Chirurgical Review for April, 1841.

therefore mention it—it is this; that, after the catamenia have entirely ceased to return, the vagina not unfrequently becomes so contracted, especially at its uterine extremity, that it is almost impervious. This circumstance should be kept in mind, in cases of suspected uterine disease, where the speculum may be had recourse to. We may here allude to another fact. Immediately before, and during the continuance of, the catamenial flow, the os uteri is found to be considerably more patent than at other times. It may therefore be of advantage, in cases of suspected polypi, to make the examination just before the usual time of its recurrence.

M. *Lisfranc* asserts with no little confidence that, when the cervix of the womb is much elongated and very conical, the woman is always barren. "If examined with the speculum, the apex is sometimes found to be so small as not to exceed a line or so across, and the os tincæ is so minute as to look like an aperture made with a small drill." He tells us that, in several such cases, he has enlarged the orifice with a *lithotome caché;* and that more than one of his patients conceived soon afterwards, although they had been long married, but had never been pregnant before. Our author very naively asks, " Will this operation be received into the domain of surgery? Experience alone can decide " *cette grande question.*" Are our readers aware of the following circumstance? M. *Lisfranc* says that the uterus, in the ordinary state, is very much more moveable than is generally imagined, and that it may be drawn down to the orifice of the vagina, in most cases requiring operations on its cervix, as in the tying of polypi, resection of cancerous growths, &c. The following are our author's own words:—

"There is a physiological fact of importance, but little known, and which hitherto has scarcely been alluded to by any writer. The uterus in the normal condition, and even when affected with engorgement, has a truly extraordinary mobility. To prove this, a very simple experiment will suffice. Let a speculum be introduced as high up the vagina as possible, so as to embrace the cervix uteri within its upper extremity; then bid the patient bear down, as if at stool; and you will perceive that, as the instrument descends, the uterus follows it, to the extent of one or even two inches—an immense advantage when the surgeon wishes to bring down the uterus to near the vulva. In cases requiring operation about the cervix, all that the surgeon has to do, is to lay hold of the os uteri with a hook and draw it gently down, until it comes fairly within sight: this may be usually effected without difficulty, and with very little inconvenience to the patient. * * * * Since the womb is thus capable of considerable descent into the vagina under the expulsive efforts of defecation, it is obvious that it must be forcibly applied against any hardened feculent matters lodging in the rectum, and therefore that any tendency to prolapsus must be much increased by straining at stool."

*Pruritus of the Vulva* is a not unfrequent and a most distressing complaint. In the treatment of it, M. *Lisfranc* places his chief reliance on an occasional bleeding from the arm. Lotions of alum, or of nitrate of silver, are sometimes very useful; but on the whole, M. *Raspail's* remedy—five parts of starch and one of camphor—is still better as a local application. It may be applied two or three times a day; the proportion of the camphor should be made to vary, according to the irritable state of the affected parts. Fumigations with the vapour of sulphur will sometimes effect a cure, when all other remedies fail. Let it ever be remembered that pruritus of the vulva is not unfrequently connected with some morbid affection of the uterus, and therefore that our chief attention must be directed to the removal of the cause. An opiate enema is sometimes a valuable remedy. M. *Lisfranc* alludes to a very obstinate case, where a very rapid cure was obtained from the administration of sulphate of quinine, after other means had quite failed. In all cases of irritation of the generative organs, M. *Lisfranc* prohibits the use of tea and coffee, more especially of the latter beverage,—which, according to his observation, exercises a marked influence on these parts. These are his words:

"My opinion on this important point of hygiene is very decided. During several years, I alternately allowed and prohibited the use of coffee to my uterine patients in the hospital; and I *invariably* remarked that, when taken, it brought on or aggravated leucorrhœal discharges, and that these abated or ceased altogether, when the use of the coffee was discontinued."

We must not attach too much importance to this remark; like all medical precepts very authoritatively laid down, it should be received with a good deal of reservation. By-the-bye, how has M. *Lisfranc* already forgotten his own very wise saying that the words *always* and *never* should be erased from medical writings? That coffee, especially if drank strong and with boiled milk, is a heating and often too an astringent beverage—boiled milk is almost always so, and is, by-the-bye, a not unfrequent cause of many children's diseases—is probably known to every one; it is therefore scarcely admissible when any inflammatory or febrile state of the system exists. But that it is injurious in most cases of leucorrhœa is a sad mistake, and one which is altogether attributable to M. *Lisfranc's* idea—certainly an erroneous one—that this discharge is almost always connected with an engorgement of the cervix uteri. For our own parts, we have by experience come to the very opposite conclusion; viz. that coffee is often a very serviceable beverage, in conjunction with other roberants, in checking leucorrhœal discharges; whereas, on the other hand, tea, being decidedly a debilitant in relaxed enfeebled states of the system, is apt to aggravate them.*

We may here introduce a remark or two on the all-important topics of the *toucher*, or the manual examination of the uterus, either per vaginam

---

* In almost all cases of protracted dyspepsia, especially when there is what is called a *craving* at the stomach, in hysterical weakness, nervous palpitations of the heart, &c., the common practice of taking large quantities of hot tea, generally twice a day, is certainly very injurious. There is, however, no set of cases in which it is altogether so hurtful as in those anomalous complaints, paralytic and neuralgic, of the nervous system, that are so common in unmarried females. We have repeatedly observed that the mere discontinuance of this favourite beverage of such patients—some of them live almost entirely upon *their tea*—has been attended with immediate benefit. The use of coffee—infused and filtered, not boiled—or weak cocoa, should be substituted in place of the trashy hot liquor that young ladies are usually so fond of. We verily believe that the use of tea has produced almost as much illness in this country as the opium, which we are so unjustly forcing upon poor John Chinaman, has among the subjects of the Celestial Empire.

or per rectum, and the use of the speculum. No one, who does not read M. *Lisfranc's* writings, can form any idea of the importance which he attaches to these means of exploration. He alludes to the tens of thousands of women whom he has examined in both ways, and frequently mentions the truly astonishing tact which he has now acquired ! His advice on this subject may be summed up in these few words : " touch, see, examine and *speculate.*"

But even, although a manual examination be made, many physicians will be unable to recognise an existing disease of the uterus. Few, according to M. *Lisfranc,* understand how to perform it properly. It is a great mistake, he says, to suppose that it is so simple an operation : he considers it to be " une des manœuvres les plus difficiles de la chirurgie," and does not hesitate to affirm that " avant nous" (*i. e.* himself) no writer has given proper instructions on the subject. He devotes an entire chapter to it. Our readers will not expect us to analyse its contents. Suffice it to say that he recommends, in certain cases where the diagnosis is obscure, that the entire hand should be introduced—" a mode of exploration by which women are not much incommoded, provided it is done with *les menagemens convenables.*"

He gives particular directions also how to examine the state of the uterus by passing the finger into the rectum, in the case of young girls ; and we shall afterwards find some remarkable discoveries which he made by following this practice.

The mere examination with the finger should rarely supersede the use of the speculum at the same time : at least, so says M. *Lisfranc,* and so French women are made to believe. Those, who imagine that the speculum may usually be dispensed with, commit a strange error—an error engendered by excessive *amour-propre et forfanterie.* He prefers the simple tubular speculum—with or without a plug—to any of the more complicated instruments that have been devised. In some cases, the orifice or the canal of the vagina may be so contracted as to render it necessary to divide the *debridement* with the scalpel, before the speculum can be introduced.

Before passing on to other topics, we select a short extract to give our readers some idea of the truly workmanlike manner in which M. *Lisfranc* sets about detecting the seat of disease; with him there is no careless trusting to merely looking at the tongue, feeling the pulse, and putting a few questions, &c. Alluding to a case of dysmenorrhœa, he says; " I practised the *toucher* on the hypogastric region, also by the rectum and by the vagina; I examined the vulva with the greatest attention; I applied the speculum; all the genital organs appeared essentially normal. I inspected the urine; I introduced a sound into the bladder; I carefully explored the ureters and the kidneys, as well as other abdominal viscera; but I could nowhere discover any morbid phenomenon."

*Frequent Mistakes in the Diagnosis of Uterine Diseases*: such is the title of the fourth chapter.

M. *Lisfranc* considers himself as, in some degree, *the* authority, " par excellence," on all questions connected with these maladies, and he therefore delivers his opinions with the *ore rotundo* of the professor who has an especial duty to watch over " l'honneur de notre noble profession."

As might be expected, he attributes a vast number of women's complaints, referred by themselves and by their ordinary medical attendants to their stomach or bowels, or mammæ, or kidneys,—to the uterus: this organ, according to him, being the " fons et origo " of almost all the ills to which they are exposed.

Some years ago, it was *gastro-enterite* on all occasions; now *l'engorgement de l'uterus* has entirely superseded, at least with our author, poor old *Broussais*' great discovery!

It will be necessary, therefore, to receive most of the statements in the present volume with no inconsiderable reservation: a *one-disease* doctor has, it is well known, always " un peu trop d'enthousiasme " on his favourite subject to be a safe authority. His description of the difficulty of persuading many women that there is any thing the matter with their womb, when their chief uneasiness happens to be seated entirely elsewhere, is really very amusing.

.. .. .. .. " When I first propose," says he, " to use the usual means of investigation, viz. manual examination and the speculum, they generally at once refuse with a sort of disdain, and leave my ' cabinet de consultation ' with the idea that I am quite mistaken in my suspicions, notwithstanding all my efforts to convince their reason and excite their fears as to the consequences. They return home; they begin to think; and now it is, when surrounded by the solicitudes of their husbands and relatives, they make up their minds to my wishes: they come back to me in a few days; apologise for their previous refusal; and seem then to sacrifice themselves to a preconceived idea:"—(what possibly is the meaning of all this?) As a matter of course, an engorgement of the uterus is discovered; proper remedies are resorted to; it is cured; and all the other *pretended* maladies of the kidneys, stomach, bowels, &c. vanish with it. Really some of the stories related by our vivacious neighbours in the present day are worthy of the pages of that doctor-mocking rogue, their own immortal author of the Malade Imaginaire.

M. *Lisfranc* enumerates the following diseases of other organs as most apt to be mistaken for engorgement of the uterus: pains in the rectum—pains in the flanks and loins—chorea—epileptiform attacks—mental alienation—hysteria—paraplegia—nymphomania—leucorrhœa—palpitations of the heart—obstinate sciatica—diseases of the bladder—pains about the umbilicus—diseases of the digestive organs—lumbago and *foiblesse des reins*—various affections of the mammæ—neuralgic headaches—amaurosis—hypochondriasis—chlorosis—dysmenorrhœa—hysteria, &c. &c. Besides these, he regards most cases of prolapsus uteri as attributable to a chronic engorgement of the organ, its increased weight being then the cause of its descent; " indeed, (says he,) we require but the simplest notion of physical laws to admit the fact, and that man must be deeply infected with medical errors who rejects it:"—a pretty summary mode of silencing all objections! Some cases are related where a full twelvemonths was required to get rid of the engorgement; but then, to make amends for this delay, the prolapsus, we are told, was cured at the same time.

To do our author justice, we must give two or three examples reported in his work, that our readers may judge for themselves of French—perhaps we should rather say M. *Lisfranc's*—practice in some of the more common diseases that fall under a medical man's notice.

The first shall be one of *Chorea.*

A girl, 18 years of age, had been affected with this malady for three years; it had resisted all "les moyens ordinaires" that had been assiduously used: (these, however, are not stated.) Menstruation had been irregular during the whole of the time. M. *Lisfranc,* on being consulted, had recourse to the treatment recommended by M. *Serres,* which has produced such *brilliant success* in a multitude of cases: from 15 to 25 leeches were applied every eight or ten days on the sides and back of the neck, and narcotic remedies were administered internally: (no treatment, by-the-bye, can possibly be worse, in most cases.) But little benefit was obtained. M. *Lisfranc* now, observing that the remedies given to act on the uterus aggravated the nervous symptoms, and hearing that his patient complained of pain and uneasiness in the pelvis, suspected that the uterus might be the offending organ.

He therefore proposed an examination by the rectum! but the proposal was rejected with "une sorte d'effroi." The attacks of chorea continued to be as frequent and severe as ever. At length the patient consented to an examination; and the uterus was found to be at least twice its normal size! An appropriate treatment was immediately commenced with the view of reducing this morbid condition of the organ; viz. occasional detraction of blood, the internal use of the ioduret of potassium, frictions on the groin and hypogastrium with the ointment of the ioduret of lead, warm baths, gentle aperients, &c. In the course of two months the engorgement of the uterus ceased, and all the symptoms of chorea had quite vanished.

The only comment, which we shall make on this report, is in the way of inquiry:—Can the size of the uterus be really discovered by the finger introduced into the rectum alone?—we ask for information.

We shall now take an instance of Paraplegia.

An unmarried woman, 45 years of age, had for six months been affected with a palsy of the lower limbs, which was attributed to a fall upon the hips. "I found," says M. *Lisfranc,* "that the catamenia had been long scanty and irregular, and that, for a considerable time before the date of the accident, the patient had experienced a sense of weight and occasionally of heat also in the pelvis. I made an examination, and found that there existed a very considerable engorgement of the uterus. The treatment, which was adopted to remove this state, proved perfectly successful, and the paraplegia also was entirely cured." How long do our readers suppose that this good maiden lady was under the doctor's hands?—only *fourteen months!* Did it never occur to M. *Lisfranc* that the paralysis in this case might possibly be owing to the effects of the contusion on the lower part of the spine? and that his usual remedies, the occasional application of leeches, friction with ioduret of lead ointment, the internal use of iodine, &c. might have acted on the seat of the injury?

The third case, that we shall give, is one of the most extraordinary—in reference to its treatment, we mean—that has come under our notice for a length of time. It is adduced as an instance of uterine disease producing palpitations of the heart!

A girl, 20 years of age, was labouring under deep ulceration of the cervix uteri, which had all the characters of cancer. It had been repeatedly cauterised, but without effect; and, as the patient's general health had begun to give way, excision of the diseased part seemed to be the sole means of saving the life of this "malheureuse femme."

The only objection to the operation was, that from the tumultuous action of the heart, and the character of the sounds heard on auscultation, it was suspected that there was an aneurysm of this organ. But as the diagnosis of this disease was considered by the doctors in attendance as not "toujours três-certain," and as patients have been known to live many years with such an affection, it was at length resolved to extirpate the neck of the womb, as affording the best chance of at least prolonging life. This was accordingly done, and no accident occurred during the operation. The severe pains, from which the poor woman had so long suffered, were, we are told, at once relieved, and on the very first night she experienced "un calme, un bien-etre qui nous frappa tous du plus vif etonnement." The palpitations of the heart speedily abated and had quite ceased by the sixth day; "le pretendu aneurisme n'existait plus." The woman entirely recovered her health, became pregnant, and was safely delivered of a living child.

As we are in an interrogatory mood, we shall suggest one or two queries about this case to our readers. Was the ulceration of the cervix uteri at all malignant? If so, was repeated cauterisation a judicious practice? Were the signs of genuine aneursym of the heart really present?; we read indeed of its "mouvements tumultueux" and of the "bruits qui appartiennent à l'hypertrophe du ventricle gauche:"—And, lastly, was excision of the os uteri necessary, or even prudent, under all the circumstances of the case?

Several chapters intervene between the chapter in which the preceding cases are recorded, and that on leucorrhœa; but, as the author seems not to have followed any obvious plan in the arrangement of his work, we need not be very particular in our selection, especially as the *refrain* of the tale is always pretty nearly the same.

*Leucorrhœa.*—The following most *lucid* exposition of the ætiology of this very common complaint is given by M. *Lisfranc.*

"This disease is produced either by a phlegmasia, or by an irritation, or by an injection, or, perhaps, by a sanguineous fluxion, which may exist, at one and the same time, in the vulva, the vagina, the internal surface of the uterus, and the ovarian tubes, whence arises a white-coloured discharge."

We verily believe that, for once that it is owing to any increase of vascular action—be the name that we give to such a state what it may—it is in nineteen, or ninety-nine cases at least, dependent upon weakness and atony of the vessels of the mucous membrane affected. Let it not be imagined that we altogether repudiate the idea that certain pale-coloured discharges from the vagina are connected with an inflamed state of the neck of the uterus: we know—and where is the medical man of any experience who does not?—that such cases are occasionally met with; but then they form the exception, and not the general rule. In what class of females is leucorrhœa most frequent?—Is it in the robust and the plethoric? or is it in those who are weakened by poor diet, by over-frequent gestation, or by excessive suckling? So common is the complaint among the lower orders in large cities, that we have no hesitation in saying that, in every dozen of middle-aged married women, there are at least eight, if not more, affected in a greater or less degree with leucorrhœal discharge.

But it is unnecessary to address arguments to the English physician on this subject, as very few are likely to be misled by the fantastic notions of many continental writers. Let us now, therefore, resume our travels through the Clinique Chirurgicale of the La Pitié Hospital Professor, who, if not a very wary or safe, is often a very amusing guide.

After the very satisfactory definition of leucorrhœa given above, our author informs us that it seldom occurs in the ovarian tubes,—a most interesting piece of information certainly, and nearly as instructive as that recorded by the traveller, that "water-mills are not often seen on the tops of mountains."

The disease, we are also told, is sometimes epidemic: we much doubt it. True, there is no good reason why the mucous membrane of the vagina and uterus may not become, in certain states of weather, the seat of irritation and subsequently of increased discharge, just in the same manner as we observe the bronchi to be affected in influenza, and the bowels in dysentery: all this is possible; but where are the trustworthy authorities for believing it?

With much greater truth, menorrhœa may be said to be *endemic* in certain places—as, for example, in all large cities—than *epidemic* at certain seasons.

The disease is aggravated, if not induced, by the injudicious use of some articles of food.

"One of the most potent causes (in Paris,) of *fleurs blanches* is, unquestionably, the use of coffee with milk." This is one of the very original remarks for which we are indebted to M. *Lisfranc*. We have already expressed our own opinions on the subject, and would not have mentioned it again, were it not to communicate "un fact très remarquable," which our author recommends to our profound meditation.

"If a woman," he says, "is in the habit of drinking her coffee pure, even although milk be taken afterwards by itself, she usually experiences no particular influence on the generative organs; but let her try the use of *Café au lait*, and ere long she will become affected with a leucorrhœal discharge." (!)

With respect to the treatment of this very obstinate complaint, there is not much novelty in the directions given by our author to comment upon.

Whenever we have reason to suspect any inflammatory condition of the uterus, it will certainly be well to take a few ounces of blood away, either from the arm, or, what is generally better, from over the sacrum by means of cupping. M. *Lisfranc* disapproves of leeches applied about the vulva, as tending, he says, to cause a derivation of blood towards the generative organs; and we believe that he is quite right here. He seems to think highly of the virtues of balsam of copaiba, and of cubebs powder—"ces precieux medicaments"—in many cases of leucorrhœa: the latter is a favourite remedy with several British practitioners, and is much recommended, if we remember aright, by Dr. *Billing* in various uterine ailments. The internal use of the ioduret of potassium is, in our author's opinion, "un moyen extraordinairement avantageux," when the disease is chronic, and the patient's constitution is not good: he employs it, he says, very frequently in his practice, and obtains effects which quite surprise him.

It is scarcely necessary to allude to any of the local remedies which are in common use; a lotion with the sulphate of zinc, or with alum, is perhaps as good as any; and if these fail, we should try the nitrate of silver. In our own (*Rev.*) practice, we have for some years past been in the habit of using the sulphate of zinc, usually along with quinine, as a tonic internally—a grain of each, in the form of pill, twice or thrice daily—and the dilute sulphuric acid at the same time. One very important therapeutic rule is to interdict the drinking of warm fluids, and more especially of tea—that favourite and most pernicious beverage of most ailing women. Shower-bathing is an admirable remedy in many cases.

*Chlorosis.*—Our author is surely not a good master at fence; else he would not expose himself so unnecessarily to the thrusts of his adversaries. The very first sentence of this chapter contains a most questionable assumption; "this disease," says he, "is most common at the age of puberty; engorgement of the uterus often determines it."

Certainly if there had been one complaint of females which one might have supposed would have escaped the *engorgement-doctrine*, it was the green-sickness of girls, and not of them only, but occasionally of boys also.

Let it not, however, be imagined that M. *Lisfranc* does not recognise the influence of other and of very different causes. This is obvious from what he says on the subject of treatment. When there is decided asthenia and marked impoverishment of the blood, he highly recommends the use of the lactate of iron; a preparation, says he, "which all practitioners now employ with such brilliant success." Bitters and other tonics may be given at the same time. If any gastritic symptoms in this exsanguined state of the system should supervene, what is the physician to do? The following extract (which we purposely give for another reason) contains M. *Lisfranc*'s opinion on this question:—

"In these difficult cases, I have seen the physicians of the Hôtel Dieu, who have always known how to keep themselves apart from the often dangerous systems of the Schools, and who have done such excellent service to our profession by inculcating the doctrines of Hippocratic medicine—(who can refrain thinking of *Horace?*

——— video meliora, proboque,
Deteriora sequor, ———)

trust almost entirely to the regulation of the food, allowing a light nourishing diet, of white meats, chicken, fish, and intermitting, for a time, the use of steel, &c. until all traces of the inflammatory symptoms have quite disappeared."

The compliment that is here paid to the medical staff of the Hôtel Dieu is, we have every reason to believe, quite merited: the names of MM. *Recamier* and *Chomel*, not to mention others—does not M. *Andral*, formerly of La Charité, now belong to this hospital?—are a sufficient guarantee for the soundness of the doctrines that are taught and the practice that is inculcated there. We have never heard of these gentlemen adopting any of the extravagant notions, that have been in and out of fashion during the last thirty years among so many of their *confreres*.

It is gratifying to us to find, that the opinion which we have formed of the leading men in Paris from the mere perusal of their writings, is entirely in accordance with that held by some of the most sensible men in the French metropolis.

The chapter on Menstruation need not detain us long; as there is little or nothing novel in it. We shall, however, make one extract, as it suggests an idea which may have escaped the attention of many a practitioner:—

"Profuse menstruation does not weaken a woman in proportion to the quantity of the blood lost; indeed, as a general remark, it seems to be true that women can bear copious hæmorrhages much better than men. They recover their strength and colour much more quickly. A man will remain pale and enfeebled for some months after a great loss of blood; but a woman, under equally favourable circumstances, will be herself again in one-half the time.

"The menstrual evacuation required that Nature should endow the female constitution with more powerful means to repair losses of blood; the hæmorrhages before and after confinement, the drain, so to speak, on the system for the nourishment of the infant, &c. demanded more efficient reparative energies.

"A practical consequence of immense importance may be deduced from these considerations. Our old masters, the physicians of the Hôtel Dieu, often impressed upon us the necessity of placing but little trust on blood-letting in the treatment of women affected with sub-inflammatory affections, in consequence of the rapidity with which losses of blood are repaired, and dwelt forcibly on the superior value of a regulated and spare diet, by which the supply of nutritive matter to the system may be kept in check. This important truth is too often quite overlooked in practice, and, in very many cases, the most judicious medical treatment is virtually rendered null by inattention to the management of the diet."

M. *Lisfranc* is perfectly right; we do not remember to have seen the remark on the difference in the reparative energies of the male and female systems so clearly insisted upon before, and we are glad of the opportunity of bestowing a modicum of commendation. Would that we could continue our praise; for indeed it is much more pleasing to all parties, critic and reader as well as author, to imitate the good example of post-prandial orators in eulogising and returning thanks to each other, than to be ever carping and finding fault. And yet what else can a man, even of the gentlest blood, do in such a case as the present? After having to wade through a hundred pages on the hacknied subjects of leucorrhœa and chlorosis, he finds that his journey is far from being at an end, and that he must submit to hear over again all the common-place remarks on dysmenorrhœa, and amenorrhœa, and retention of the catamenia, and menorrhagia, &c. &c. As we have already asked before, what have all these diseases to do with a clinical course of surgery? True, there may be a ward for female complaints in the Hôpital de la Pitié, and crowds of women may flock weekly, as we are told they do, to the consultation of M. *Lisfranc*, where, " in the sacred cause of humanity," they are *touched* and *speculated* with the most pains-taking care; but still we contend that, using language in its ordinary acceptation, all this cannot strictly be called surgery; and that it is not " Clinique de l'Hôpital de la Pitié" is proved by the circumstance of most of the cases reported having been derived from the author's private practice, and certainly not from the wards of the hospital whose name is here given.

Even this, and much more, we would have most willingly overlooked, had there been some really valuable matter communicated; but when the burden of the whole is the ever-recurring words, engorgement of the uterus, we verily believe that the patience of the man of Uz himself would have been sorely tried, had he been a medical reviewer. Judge, pensive reader, for thyself: here is an extract from the chapter on menorrhagia;—

" In certain circumstances, especially, we must ascertain by manual examination, and also with the speculum, if the blood comes from the uterus. The *toucher* must also be practised to determine the state of this organ; as idiopathic or essential metrorrhagia is much less common than is generally imagined. I have proved, at the public consultations of our hospital, that, of 50 patients affected with abnormal discharges of blood of a month's continuance, in 46 there is some morbid affection of the womb—either an engorgement or a mole, or hydatids, or a fibrous tumor, or ulcers: in a few cases the ovaries alone are diseased. I have uniformly insisted much at my clinique on this great and important truth. If it be neglected, medicine becomes merely a catalogue of symptoms, and but little success can be expected to attend the labours of the physician. I have elsewhere shewn, by numerous facts, that uterine hæmorrhage in many cases cannot be checked until the disease of the uterus, on which it depends, be relieved or cured. Once more, I repeat, *touchez donc dans tous les cas;* I cannot too earnestly insist upon this precept; and do not imitate, I beseech you, the example of, alas! too many physicians, who, for months, nay for years, will sometimes attend patients, without once thinking of this most potent means of exploration."

When will a phlegmatic Englishman ever shew half such *empressement* as this in enlightening his readers? and all to urge them to use the *toucher* and the speculum more frequently than they are in the habit of doing. So indispensable does M. *Lisfranc* consider these means, that, as if to give them precedence over every thing else in the history of uterine complaints, he actually commences his description of " the nervous states of the womb," with these remarkable words: " On touche; on applique le speculum et l'on trouve la matrice à l'etat où nous l' avons vue dans le chapitre precedent!" This (the preceding) chapter, by-the-bye, is headed *Malaises de l'Uterus;* and, as it stands by itself, apart on the one hand from the nervous and on the other from the inflammatory affections of the organ, we are somewhat puzzled what to make of it. This, however, we learn from M. *Lisfranc* that, if we practise the *toucher* in this disease, the neck of the womb will be found dilated, as it usually is, for a few days before and after the catamenia, and that the application of the speculum will shew that the os tincæ is "très-legerement fluxionné."

Even in cases of hysteria, we are recommended by our enthusiastic author to adopt the same methods of examination; as, in not a few instances, the symptoms of this most anomalous complaint are attributable to uterine disease as their cause.

All that we can say on this head is, that we sincerely trust that British medical men will never so far forget the respect that is due to common decency, as to be led to imitate the advice that is so strongly urged in the present work.

If we remember aright, there was a book published in this country, two years or so ago, by a Mr. *Jones* who had been infected with an admiration of French practice, and who very gravely informed us that, with a little delicate management on the part of the physician, there is scarcely any woman in England who will be found to object to the employment of the speculum. He deserves to have his back well flayed with *Christopher North's knout*—does he know what that is?—for saying so. Heaven forbid that, with other fashions from Paris, we should ever import our notions of decorum and morality from that gay and licentious metropolis.

A Frenchman's idea of decency is, in many respects, very different from our own; almost the whole of their modern literature—in spite of some glorious exceptions; witness *Chateaubriand, Lamartine,* and *Guizot*—is of a loose, or positively filthy stamp; and it is, therefore, perhaps not wonderful that their medical writers have not escaped the prevailing spirit of the times. Certain it is that many of the details in this work of M. *Lisfranc* ought never, in our opinion, to have been made public; at least we know that, if published in this country, under the guise of scientific or professional reports, they would meet with universal reprobation. For example, not only have we an express chapter, of between 20 and 30 pages, on the subject of "nimia salacitas" in women, and elaborate instructions how to treat it in patients of all ages, from the young girl to the old worn-out harridan; but in a subsequent chapter we have minute directions how the physician ought to act, about forbidding sexual intercourse between men and their wives. Remember, however, reader, that M. *Lisfranc* on this, as on every other occasion, is "toujours guide par les nobles sentiments de notre belle profession." It may be so; we do not doubt his word; but strange certainly are the lengths to which his fancy carries him.

In his enthusiastic pursuit of engorgement of the uterus, he allows no consideration to put him off the scent, if he once suspects that the crafty foe is burrowing there. The following extract will shew our readers what we mean better than any exposition of our own.

"There are women who seem to enjoy capital health, who are fat and fair, and in whom the uterine functions are performed quite normally; yet these unfortunate creatures, living though they are in the most complete security, if interrogated by a sage physician will acknowledge that they sometimes experience a sense of weight and of heat in the pelvis, perhaps that they cannot stand long erect, and that they feel weak in the loins. Most probably if they happen to speak to a medical man, he tells them that there is little or nothing the matter, and that no serious mischief can exist with such brilliant health as they have, especially as there may be no discharge from the vagina, either white or red-coloured.

"Be on your guard! my friends; you may commit a most dangerous mistake, if you do not make a manual examination, and if you do not apply the speculum; for very probably there exists a disease, which ere long will suddenly break forth with the most alarming symptoms of the most confirmed cancerous diathesis. Such cases are not unfrequent; I meet with two or three every month; the sacred interests of humanity compel me to say that these unfortunate women are the victims of medical ignorance."

Can mere enthusiasm go farther than this? nay, does it not trespass on the limits of insanity? So entirely is our author's whole mind occupied with the subject of engorgement of the uterus, that more than once it has been suspected that he is the victim of a monomaniacal delusion; and now that the *corps de sa maladie*—as his present work may very justly be considered —has been made public, the suspicion in question has certainly derived not a little confirmation. No mind, it has been reasonably imagined, that is perfectly sane could allow one prevailing idea to obtain and keep such a mastery over it, as that of an enlarged womb has acquired over the mental operations of M. *Lisfranc;* but then it has been asked, "If so, what is the particular faculty, and what is the phrenological organ, that is mainly at fault?" Here certainly lies the difficulty in the ultimate diagnosis of the malady—for malady there *must* be, in spite of the brilliant health the patient seems to enjoy. Some have fancied that the organs of *Marvellousness* and *Ideality* may have become congested, or that those of *Caution* and *Causality* have been undergoing a partial atrophy; while others have thought that *Individuality*, and some of the more perceptive faculties, must be chiefly affected. At first we hesitated a good deal between these opinions; but, on a more mature consideration of the history of the case, a new light has flashed across us; we begin to suspect that the *cerebellum* is the offending organ, and that, from some cause or another, this part of the nervous system has become the seat of a "fluxionary congestion" of its vessels. We may term the disease *utero-mania* for two reasons; in the first place, it specifies distinctly the object of the monomaniacal delusion, the real *causa peccati;* and, in the second, it is highly probable that the part chiefly affected is what Holofernes in the play (Love's Labour Lost) has so happily designated as "the womb of the pia mater," and "the ventricle of memory."

Now for the treatment: fortunately this is very obvious. Acting upon the principles, which M. *Lisfranc* has so earnestly inculcated in his present work, we have recommended that a *spoliative* bleeding be taken from the arm occasionally,—say once a month during the first quarter,—and afterwards less frequently; that the cupping-glasses, both *scarifiées et seches*, be applied over the nape of the neck in the intervals between the venæsections; that cold lotions be kept constantly on the occiput; that the hydriodate of potash be given internally, and the ointment of ioduret of lead be used externally; and that the patient be kept on a spare diet, avoiding particularly *café-au-lait*. Along with these medicinal and dietetic regulations, we would suggest, in as delicate a manner as possible, the propriety of his *living quietly* apart, and avoiding whatever may be likely to excite or exhaust his cerebellum. We observe that he has announced his intention of speedily publishing a third volume of his Clinique Chirurgicale: we strongly dissuade him from doing this for at least a twelvemonth. By that time, the treatment now recommended, if persevered in steadily, will, we are confident, have worked a most salutary change in the state of his malady.

